# Prevalence of blood-borne viruses and hepatitis B vaccination status among haemodialysis patients in Central Australia

**DOI:** 10.1016/j.ijregi.2022.09.010

**Published:** 2022-09-27

**Authors:** Arun Prakas Ramaswami, Basant Pawar, Gitanjali Pawar, Megan Brown

**Affiliations:** 6 Gap Road, Alice Springs Hospital

**Keywords:** Blood-born viruses, Hepatitis B vaccination, Haemodialysis

## Abstract

**Objective:**

A cross-sectional study to determine the prevalence of blood-borne viruses and hepatitis B vaccination status in haemodialysis patients in Central Australia.

**Methods:**

Our study comprised 366 Aboriginal and 1 non-Indigenous Australian in Central Australia who had commenced haemodialysis between January 1996 and December 2019.

**Results:**

Chronic hepatitis B infection was seen in 8.4% of patients, and serological evidence of human T-lymphotropic virus 1 in 28.3% of patients. The prevalence of HIV and hepatitis C was less than 1%. The vaccine status of all 182 patients who had received the hepatitis B vaccine was reviewed. Vaccine response was seen in 72.2% of patients who had received the vaccine at birth or in early childhood. There were 99 patients aged 20 years and older who had received hepatitis B vaccines before their haemodialysis commenced. Vaccine response was observed in 88.9% of these patients. A seroconversion rate of 78.5% was achieved in vaccine naïve patients who received the hepatitis B vaccine after their haemodialysis commenced.

**Conclusion:**

The response to the hepatitis B vaccine among haemodialysis patients in Central Australia was suboptimal and variable. The prevalence of chronic hepatitis B infection declined after the universal hepatitis B vaccination was introduced in 2000.

## Highlights

• High prevalence of HBV and HTLV-1c infections
• Low prevalence of other BBV infections in Central Australia haemodialysis patients
• No patients with chronic hepatitis B infection were less than 40 years old
• Hepatitis B vaccination status of the Indigenous population in Central Australia
• The response to the hepatitis B vaccine was suboptimal and variable

## Background

The Northern Territory has the highest prevalence of haemodialysis-dependent end-stage kidney disease in Australia at 2956 cases per million population compared with the Australian prevalence of 536 per million (ANZDATA. The 42nd Annual ANZDATA Report 2019). The Central Australian renal services' catchment area encompasses two-thirds of the Northern Territory with a land mass of 872,861 square kilometres and a population of 48,506 people (Department of Health, Northern Territory 2022).

Chronic hepatitis B virus (HBV) infection is defined by the continued presence of hepatitis B surface antigen (HBsAg) in the blood for longer than 6 months (Krajden et al., 2005). The prevalence of chronic HBV in Australia is highest in the Northern Territory (1.7%) and lowest in the state of Tasmania (0.67%) (MacLachlan et al., 2018). The prevalence of chronic hepatitis B in patients undergoing haemodialysis in Western Europe and the United States ranged from 0% to 7% (Covic et al., 1999) in a 1999 report, with a higher prevalence in developing countries than in developed countries (2%–20%) (Fabrizi et al., 2008)^.^ Interestingly, in haemodialysis patients, occult HBV infection, defined as the presence of detectable HBV DNA in HBsAg-negative individuals with the potential risk of transmission during haemodialysis, reportedly occurs at a rate of 1.3% to 3.8% (Minuk et al., 2004).

The hepatitis B vaccine is effective in preventing hepatitis B infection. The universal program of vaccinations at birth began in Northern Territory in 1990, followed by a catch-up program in 1998 targeting 6- to 16-year-olds. Those born between 1982 and 1988 were eligible for this catch-up program. In 2000, the Australian government implemented a universal infant/adolescent hepatitis B vaccination program for newborns and adolescents; they received Engerix B (10 µg) at 0, 2 and 6 months. The Australian Technical Advisory Group on Immunisation (ATAGI) has recommended reviewing the hepatitis B vaccination status of all Aboriginal people and providing hepatitis B vaccination to non-immune Aboriginal people aged 20 years and older, as they have a higher risk of acquiring new hepatitis B infections than non-indigenous people (Wattiaux et al., 2016). They received Engerix B (20 µg) at 0, 1, 2 and 12 months. In addition, adult haemodialysis patients or those with chronic kidney disease stage 4 or 5 are recommended to receive larger than usual doses of recombinant hepatitis B vaccine, consisting of 40 µg of the H-B-VAX II dialysis formulation at 0, 1 and 6 months (Babu and Kotton, 2021).

The human T-lymphotropic virus type 1 (HTLV-1) is an oncogenic retrovirus that infects CD4+ T-cells, and transmission follows exposure to infected lymphocytes during blood transfusions, breastfeeding or sexual intercourse (Einsiedel et al., 2016, Einsiedel and Pham, 2016). HTLV-1 infection is endemic in Central Australia with a positive rate that varies geographically from a regional high of 51.7%, declining with distance from central Australia, with a lower prevalence in the Top End of the Northern Territory (0.6%–2.2%) (Davies and Jabbar, 2012, Grivas et al., 2014). A review of a hospitalised population in Central Australia reported that 19.2% of patients with HTLV-1 tested positive for HBsAg (Einsiedel et al., 2014). Aboriginal and Torres Strait Islanders are disproportionately affected by hepatitis C infection (HCV), with a notification rate in 2016 in this population 3.8 times higher than in the non-Indigenous population (The Kirby Institute 2017). Similarly, notifications of HIV in 2016 were more than 3 times higher among Aboriginal and Torres Strait Islander women than among Australian-born non-Indigenous women (1.1 vs 0.3 per 100,000); in the 10 years from 2007 to 2016, population rates of newly diagnosed HIV per 100,000 in the Northern Territory varied between 2.6 and 8.1 (The Kirby Institute 2017).

The aims of this study were: (1) To determine the prevalence of blood-borne virus infections, in particular hepatitis B infections, in a haemodialysis population in Central Australia; (2) To determine the hepatitis B vaccine response in those cohorts of haemodialysis patients who received the vaccine before and after haemodialysis commenced; (3) To evaluate the factors that could affect the responsiveness of hepatitis B vaccine in vaccine naïve patients who have already commenced haemodialysis. All previous studies on HTLV-1 and HBV infections in the Northern Territory were community-based except one in 2010 which looked at haemodialysis patients in the Top End of Australia (Davies and Jabbar, 2012). In our study, the prevalence of blood-borne viruses in haemodialysis patients in Central Australia was compared with that of a similar cohort from the Top End of the Northern Territory. Our study is significant regarding hepatitis B vaccine response in haemodialysis patients as it is the largest study ever conducted and the only one with a predominantly Aboriginal cohort.

### Methodology

Our cross-sectional study included all 367 patients on maintenance haemodialysis at 3 large haemodialysis units in Alice Springs and Tennant Creek. The patients had commenced haemodialysis between January 1996 and December 2019. Patients receiving peritoneal dialysis and renal transplant recipients who were not on any dialysis with stable graft function were excluded from the study. All patients were tested for HBsAg, hepatitis B total core antibody (HBcAb), hepatitis B surface antibody (HBsAb), HTLV-1, HCV and HIV serology at the start of haemodialysis. Their clinical data and serology results were collected from the clinical and laboratory electronic reporting system for the Northern Territory. Information available in the primary care information system, a client-focused health information system tailored for Northern Territory remote health centres, was also reviewed. The HBV, HIV, HCV and HTLV serology results from the start of haemodialysis and the most recent retests were reviewed, and any changes between them were noted.

Patients with isolated HBcAb positive results underwent HBV DNA testing every 6 months to detect occult HBV infection. HBsAg, HIV and HCV serology follow up tests were performed every 6 months. All haemodialysis patients were tested yearly for evidence of HBV immunity with HBsAb titre. When HBsAb titre dropped to less than 10 IU/mL, patients received a booster dose. All patients in the study who had commenced haemodialysis and were non-immune at the time dialysis commenced received the H-B-Vax II dialysis formulation (40 µg) in a 3-dose schedule at 0, 1 and 6 months. The response was determined by measuring HBsAb at 6 weeks after the last dose. Factors that might affect the responsiveness to hepatitis B vaccination, such as age, gender, coexistence of HTLV-1 infection, serum parathyroid hormone and serum albumin, were also evaluated in this study.

HTLV-1 serological status was determined in 2008 using a particle agglutination assay (Serodia-Fujirebio Inc., Tokyo, Japan) and since 2009 via an enzyme immunoassay (Abbott Architect rHTLV I/II assay). Reactive and indeterminate serology results were sent to the National Serological Reference Laboratory for review using the western blot method. The HCV assay currently being used is the VITROS Anti-HCV assay; previously, the Abbott architect anti-HCV assay was used. The HIV serology assay is the Abbott Architect HIV Ag/Ab combo assay. HBV serological assays are currently performed using the VITROS immunometric immune assay HBsAg, the HBsAb, and HBcAb assays; the Abbott Architect assay was previously used. The primary assay used from mid-2011 until November 2019 for HBV viral load was Roche COBAS® AmpliPrep/COBAS® TaqMan® HBV Test, version 2.0. Before that, the primary assay was the Bayer Versant HBV DNA 3.0 assay, which had a lower limit of quantification of 351 IU/mL. As these tests were repeated at regular intervals, serological results with the latest assay were available for all patients.

### Statistics

Descriptive statistics reported included counts and proportions for categorical variables, while mean, median, standard deviation, minimum, 1st quartile, 3rd quartile and maximums were reported for continuous variables. Association testing between categorical variables was performed using χ2 and Fisher's exact tests. Testing of continuous variables across the two groups was performed utilising a two-sample t-test. P<0.05 was considered statistically significant. A multivariate regression model was used to predict HBsAg positivity, with age, gender and HTLV-1 as variables. The same model was used to predict HTLV reactivity according to age and gender. For comparison of HTLV-1 infection between men and women, a t-test was implemented.

## Results

### Demographic Characteristics

Of the 367 patients receiving haemodialysis in Central Australia, only one was non-Indigenous; the others were Aboriginal Australians. Most of the patients were women (60.8%). Age at the commencement of dialysis ranged from 23 to 84 years, with a mean age of 53.1 ± 11.5 years and a median of 53. The average duration of haemodialysis treatment was 5.83 years (standard deviation: 5.83 ± 4.53). One patient was diagnosed with HCV infection. No patients tested positive for HIV infection before or after haemodialysis commenced.

### Hepatitis B virus

Overall, 41.9% (154/367) of patients showed evidence of past hepatitis B virus infection (HBcAb-positive, HBsAg-negative ± HBsAb >10 IU/mL), including patients with isolated positive HBcAb results and those with resolved HBV infection. A total of 128 (34.9%) patients were immune to hepatitis B infection through naturally acquired immunity with elevated HBsAb titres and were HBcAb positive. None of these patients received a hepatitis B vaccination at any point in time. The viral load was undetectable in all 26 (7%) patients who showed isolated HBcAb positivity. There were no cases of occult hepatitis B infection in the study group. Two patients with a history of resolved hepatitis B infection continued to receive immunosuppressive therapy for vasculitis and were followed up regularly (Table 1, 2).

**Table 1:** Hepatitis B profile of the haemodialysis patients in Central Australia

Hepatitis Markers	HBcAb+ & HBsAb+	HBsAg+ & HBcAb+	Isolated HBcAb+	HBsAg– & HBsAb–	HBsAg–HBcAb– HBsAb+	Total
**Sex**						
Male	60	16	9	7	53	143
Female	68	15	17	23	99	224
Total	128	31	26	30	152	367
Age						
<40	10	0	2	8	23	43
41-50	40	7	8	10	35	98
51-60	46	13	7	8	55	129
61-70	25	10	7	3	27	72
71-80	7	1	2	1	12	25
**HTLV**						
Reactive	33	14	6	4	47	104
Negative	95	17	20	26	105	263

(1) HBcAb+ & HBsAb+ =Immune due to natural infection, (2) HBsAg+ & HBcAb+ = chronic hepatitis B infection, (3) isolated HbcAb positive = Occult hepatitis B infection, window period or recovering from acute hepatitis B infection, (4) HbsAg– & HbsAb– = patients not responded to hepatitis B vaccine (5) HBsAg – & HbcAb– & HbsAb+ = patients responded to hepatitis B vaccine.

**Table 2:** Demographic profile of haemodialysis patients in Central Australia

Age range (years)	<40	41-50	51-60	61-70	>71	Number (%) Or mean ± SD
**Sex**						
Male	18	34	60	27	4	143 (39.2)
Female	26	64	68	43	22	224 (60.8)
**Renal disease**						
Diabetes	20	56	70	50	15	211 (57.5)
Diabetes + Hypertension	5	24	28	14	6	77 (20.9)
Glomerulonephritis	15	10	20	2	1	48 (13)
Reflux Nephropathy	4	6	6	4	3	23 (6.3)
Unknown	-	-	-	-	-	8 (2.2)
**HTLV-1 Reactive**	8	21	43	26	6	104
**Haemodialysis vintage** (in years)	5.83	5.84	5.85	5.84	5.93	5.83 ± 4.53
**On Immunosuppression** (For vasculitis)	1	3	2	0	0	6

Thirty-one (31/367, 8.4%) patients showed evidence of chronic hepatitis B infection and were HBsAg positive at the time dialysis commenced. The mean age of patients positive for HBsAg was 56.7 ± 8.3 years (range: 42–71), while that of patients negative for HBsAg was 52.8 ± 11.6 years (range: 23–84); the difference in ages was statistically significant (t = 2.403; p = 0.02). Of these 31 patients, 29 were in the immune control phase with HBV DNA levels less than 20 IU/mL. Five patients in the immune control phase also had cirrhosis and were treated with entecavir. One patient who was positive for hepatitis B e antigen (HBeAg) with a high viral load was in the immune clearance phase of hepatitis B and was also treated with entecavir. The precore mutant variant ‘e’ antigen marker was negative in another patient who was in the immune escape phase. The patient died due to the rapid progression of liver disease and hepatocellular carcinoma. All patients with chronic hepatitis B infection were dialysed using dedicated dialysis machines, and there were no cases of hepatitis B infection directly attributable to haemodialysis as none developed HBsAg during haemodialysis.

### Human T-lymphotropic virus-1

Serological evidence of HTLV-1 infection was obtained through positive western blot results in 104 of the 367 patients. Of the 104 patients reactive for HTLV-1 infection, 65 were women and 39 men. Multivariate regression analysis for predicting HTLV reactivity showed that age was significantly associated with HTLV-1 infection and every 1-year increase in age increased the odds of HTLV-1 infection by 2% (p = 0.03). Because a regression model was used for the analysis, an association was identified between age and HTLV-1 infection, but the causative effect of age on HTLV infection was not able to be analysed (Table 3). No association was found between gender and HTLV infection status (women vs men, 62.5% vs 37.5%, p = 0.8). HBsAg positive patients had a higher HTLV-1 reactivity rate (45.2%) than that of HBsAg negative patients (26.8%, p < 0.05). Multivariate regression analysis for predicting HBsAg positivity with age, gender and HTLV-1 infections as variables revealed that when controlled for age and gender, HTLV-1 infection was significantly associated with HBsAg (odds ratio: 2.25; 95% CI: 1.07–4.54; p = 0.03) (Table 4).

**Table 3:** Multivariate regression model for predicting HTLV reactivity from age gender

HTLV1	Reactive n=104	Non-Reactive n=263	Odds ratio (95% CI)	p-value
**Age** (SD)	55.2 (10.3)	52.3 (11.8)	1.02 (1 - 1.04)	0.03
**Sex**				
Male n (%)	39 (37.5)	104 (39.5)	0.92 (0.57 – 1.46)	0.8
Female n (%)	65 (62.5)	159 (60.5)		

Number in data frame = 367, Number in model = 367, Missing = 0 AIC = 438.8, C-statistic = 0.583, H&L = Chi-sq. (8) 8.30 (p=0.405) MODEL FIT: χ² (2) = 4.76, p = 0.09, Pseudo-R² = 0.02

**Table 4:** Multivariate regression for prediction of HBsAg positivity from age, sex, and HTLV-1.

HBsAg	Positive n=31	Negative n=336	Odds ratio (95 % CI)	p-value
**Age (SD)**	56.7 (8.3)	52.8 (11.7)	1.03 (1.0 – 1.07)	0.08
**Sex**				
Male n (%)	15 (48.4)	128 (38.1)	1.52 (0.72 – 3.19)	0.26
Female n (%)	16 (51.6)	208 (61.9)		
**HTLV-1**				
Reactive n (%)	14 (45.2)	90 (26.8)	2.25 (1.07 – 4.75)	0.03
Non-reactive n (%)	17 (54.8)	246 (73.2)		

Number in data frame = 367, Number in model = 367, Missing = 0 AIC = 211.8, C-statistic = 0.663, H&L = Chi-sq. (8) 8.88 (p=0.353) MODEL FIT: χ²(2) = 8.74, p = 0.03, Pseudo-R² = 0.05

### Immune response to Hepatitis B vaccine

Of the patients on maintenance haemodialysis, 152/182 (83.5%) responded to the hepatitis B vaccine with HBsAb levels >10 IU/mL. Thirty (16.5%) patients did not respond to the hepatitis B vaccine and were considered non-responders. Among the 152 patients who responded to the vaccine, 108 (71%) had an adequate response as evidenced by HBsAb levels >100 IU/mL and the remaining 44 (29%) patients were seroconverted, with lower titres; these patients received hepatitis B vaccine at different times in their lives.

Of the 182 patients on maintenance haemodialysis, 65 received the vaccine after the haemodialysis commenced; 51 (78.5%) of these responded to the vaccine, while 14 (21.5%) were non-responders (Table 5). Patients who failed to develop adequate HBsAb titres after a full vaccination course were given another course with doses at the same intervals. Those who failed to seroconvert after 2 courses (6 injections) were categorised as non-responders. Responders and non-responders had a mean age of 56.60 ± 10.1 years and 56.62 ± 15.9 years, respectively (p = 0.8). An analysis performed to detect any association between the antibody response and gender, HTLV, serum albumin and serum parathyroid hormone (iPTH) found no differences between responders and non-responders for these factors. A similar response rate to vaccine was observed in the haemodialysis population with or without diabetes mellitus (82.3% vs 71.4%, respectively; p = 0.4) who were either type 1 or 2.

**Table 5:** Comparison between responders and non-responders in vaccine naïve patients

Vaccine	Responders n=51	Non-responders n=14	p-value
Age in years (SD)	56.60 (10.1)	56.62 (15.9)	0.8
Sex			
Male n (%)	15 (29.4)	3 (21.4)	0.7
Female n (%)	36 (70.6)	11 (78.6)	
Diabetes			
Yes	42 (82.3%)	10 (71.4%)	0.4
No	9 (17.7%)	4 (28.6%)	
HTLV-1			
Non-reactive n (%)	38 (74.5)	13 (92.9)	0.2
Reactive n (%)	13 (25.5)	1 (7.1)	
iPTH (pmol/L mean ± SD)	48.8 (26.3)	50.6 (34.7)	0.8
Serum albumin (g/L mean ± SD)	37.4 (5.2)	35.5 (4.1)	0.2

1.Diabetes -either type 1 or type 2 diabetes mellitus 2. iPTH- parathyroid hormone

Among the remaining 117 patients who received the hepatitis B vaccine before haemodialysis commenced, 18 were dialysis patients who received the vaccine either at birth or in early childhood through universal immunisation and catch-up programs between 1989 and 2001. Twelve of these patients received the vaccine at birth, and 6 received the vaccine as part of a catch-up program. Vaccine response was observed in 13 of these 18 patients (72.2%). The remaining 5 patients (27.8%) had HBsAb levels less than 10 IU/mL even after another 2 full courses of the vaccine; it is uncertain whether an initial response was seen in these 5 patients after vaccination at birth since testing for vaccine response was not done routinely. One patient among the responders and 2 among non-responders received the vaccine before 1991, when a plasma-derived vaccine was used. The remianing ninety-nine of the 117 patients were adults who received the hepatitis B vaccine before haemodialysis commenced; 88.9% (n=88/99) of these patients responded to the vaccine with raised HBsAb levels >10 IU/mL (Table 6).

**Table 6:** vaccine response at different times in life before and after starting haemodialysis

Timing of vaccine Response n (%)	At Birth and adolescents	Before starting haemodialysis	After starting Haemodialysis
Responders	13 (72.2)	88 (88.9)	51 (78.4)
Non-Responders	5 (27.8)	11 (11.1)	14 (21.6)
Total	18	99	65

## Discussion

Patients on maintenance haemodialysis for end-stage renal disease commonly experience blood-borne viral infections (Davies and Jabbar, 2012). Our study shows that HBV prevalence in haemodialysis patients in Central Australia was comparable to HBV prevalence in haemodialysis patients at the Top End of the Northern Territory (41.9% vs 42.7% HBcAb positive: 8.4% vs 8.9% HBsAg positive) (Davies and Jabbar, 2012). HIV and hepatitis C prevalence were similarly low (<1% vs 1.6%).

The prevalence of chronic HBV (8.4%) falls within the “highly prevalent category” according to the criteria described by the World Health Organization (MacLachlan and Cowie, 2015) and it is likely that HBV infections occurred at birth or early in life for many of the patients in this study. No patients with chronic HBV infection were under 40 years old; 7 were 41–50, 11 were 51–60, and 13 were 61–70 (Table 1). This age profile indicates that the prevalence of chronic HBV infection declined after the introduction of the universal hepatitis B vaccination in 2000. Previous studies have shown that the prevalence of chronic HBV in the Northern Territory was 0.8% after the introduction of the hepatitis B vaccine, whereas it was 14.2% for those born in the pre-vaccination era (before 1988) (Graham and MacLachlan, 2019).

HTLV-1 seropositivity was observed in 28.3% of patients undergoing haemodialysis in Central Australia. In contrast, only 2.2% of patients undergoing haemodialysis at the Top End of Australia showed HTLV-1 seropositivity (Davies and Jabbar, 2012). This high prevalence of HTLV-1 positivity in patients on haemodialysis in Central Australia is generally attributed to the inclusion of patients from different communities where the seroprevalence varies from 12.6% to 61% (Einsiedel et al., 2016) (Einsiedel and Pham, 2016). The rate of HTLV-1 infection increased with age, consistent with previous studies in Central Australia (Einsiedel et al., 2016). There was no association between gender and HTLV infection status. Patients with chronic HBV had a higher prevalence of HTLV positivity than patients who were HBsAg negative (p=0.04) (Table 4) which could be attributed to sexual transmission of both blood-borne viruses, as has been reported in previous studies conducted in Central Australia and the Northern Territory (Marr et al., 2017, Turpin et al., 2019).

A seroconversion rate of 78.5 % (n= 51/65) was achieved in 65 vaccine naïve patients who received the hepatitis B vaccine after haemodialysis commenced. The remaining 21.5% (n=14/65) were non-responders, as they failed to seroconvert even after 2 full courses of vaccine with doses at the same intervals. In these patients, chronic HBV was excluded as one of the causes of vaccine failure; this response in haemodialysis patients was suboptimal compared with that of the general population. The response to the hepatitis B vaccine is approximately 90% in the general population (based on studies conducted on healthcare workers who represent the general population (Chathuranga et al., 2013)). Few studies have been conducted on the vaccine response in haemodialysis patients. In a study by Miłkowski et al., the efficacy of the hepatitis B vaccination was found to be 77.5% in haemodialysis patients (Miłkowski et al., 2000). Peces et al. reported a seroconversion rate of 77.5% 1 month after vaccination in 80 seronegative haemodialysis patients (Peces et al., 1997). In our study, no statistically significant difference was observed between responders and non-responders with regard to age, gender, HTLV-1 infection, serum albumin level and iPTH level. A similar response rate to the vaccine was observed in our cohort of patients on haemodialysis with or without diabetes; however, some studies have reported that patients on haemodialysis with diabetes respond less favourably to the vaccine (Chin, 2003).

We observed vaccine response in 13 (72.2%) patients who received the vaccine at birth or in early childhood, including those who participated in universal immunisation and catch-up programs. Studies have shown that immune responses to early hepatitis B vaccination may have been suboptimal in some Aboriginal communities, as plasma-derived vaccines were used before the introduction of recombinant vaccines in 1991. Plasma-derived vaccines, when used in infancy, were accompanied by a relativity inferior anamnestic response (Dent et al., 2010). Although most participants responded to a booster dose, the significance of the increased proportion of non-responses among older adolescents might indicate waxing immune memory (Samandari et al., 2007). The remaining 99 patients who were aged 20 years and older when they received the hepatitis B vaccination, were vaccinated at different times in their lives before they commenced haemodialysis. Vaccine response was observed in 88.9% (n=88/99) of these patients. Hepatitis B vaccination of Aboriginals aged 20 years and older was based on the recommendation of ATAGI as they were at a higher risk of acquiring new hepatitis B infections than non-Aboriginal people (Wattiaux et al., 2016). This vaccine guideline has had a positive influence on the prevention of hepatitis B infection in Aboriginal people, especially in patients with chronic kidney disease, as previous studies had shown that patients with higher glomerular filtration levels were more likely to respond to hepatitis B vaccination with seroconversion, independent of other factors (DaRoza et al., 2003).

The limitations of our study are its retrospective nature and small sample size. The association between HBV infection and HTLV-1 is epidemiologically expected, although the weight of observation was decreased in this study by the low sample numbers. As this was a retrospective study, limited data were available on factors associated with a risk of HTLV infection, such as blood transfusion history and sexual history; hence risk factor identification was not performed. The full impact of our study with respect to universal vaccinations was difficult to assess as the mean age of haemodialysis patients was 53.1 ± 11.5 years. Factors affecting the effectiveness of the hepatitis B vaccine in Aboriginal people who received the vaccine before haemodialysis commenced were not considered in this study as this was a retrospective study and there were limited data available regarding factors such as chronic kidney stage, diabetes status or the presence of HTLV- 1 infection at the time the vaccine was administered.

## Conclusion

High prevalence rates of HBV and HTLV-1 and low prevalence rates of other blood-borne viruses were observed in patients undergoing haemodialysis in Central Australia. The prevalence of HBV infection has dramatically declined since the introduction of universal compulsory vaccination. The response to the hepatitis B vaccine among those undergoing haemodialysis was suboptimal and variable. Based on ATAGI guidelines, it is recommended to administer hepatitis B vaccines to the Aboriginal population aged 20 years and older if they are found not to have immunity to the hepatitis B infection.
